# A Rare Case of a Giant Choledochal Cyst in a Caribbean Infant

**DOI:** 10.7759/cureus.57735

**Published:** 2024-04-06

**Authors:** Paige V Ali, Fiaz Ali, Sarita Sudama, Lakhan Roop

**Affiliations:** 1 Paediatric Surgery, San Fernando Teaching Hospital, San Fernando, TTO

**Keywords:** obstructive jaundice, paediatric abdominal masses, hepato-jejunostomy, paediatric surgery, hepato-biliary, choledochal cysts

## Abstract

Choledochal cysts are uncommon dilatations of the biliary tree. Giant choledochal cysts are those that exceed a maximum diameter of 10cm. Our case describes a female infant who presented to our paediatric surgery department with a three-day history of vomiting, abdominal distention, pale stool, and irritability. On palpation, she was found to have a large abdominal mass and the computed tomography (CT) scan showed a giant choledochal cyst. The patient underwent laparotomy with cholecystectomy, choledochal cyst drainage and complete excision, with hepaticojejunosotomy. At the last follow-up three years post-surgery, all growth parameters and liver enzymes were within normal ranges. To the best of our knowledge, this is the first documented case of a giant choledochal cyst in the paediatric Caribbean population.

## Introduction

Choledochal cysts are rare congenital abnormalities of the biliary tree [1]. They usually present in paediatric patients. Giant choledochal cysts measuring ≥10cm are even more uncommon. Documentation of such in Caribbean literature is scarce. The Todani classification is used to identify the various types of choledochal cysts and management varies based on cyst classification [2]. Type 1 encompasses the most frequently occurring choledochal cyst [3]. The case presented below details the detection of a Type 1 giant choledochal cyst in a three-month-old female, and the surgical and clinical management given to this patient at the San Fernando General Hospital, Trinidad and Tobago.

## Case presentation

**Figure 1:**
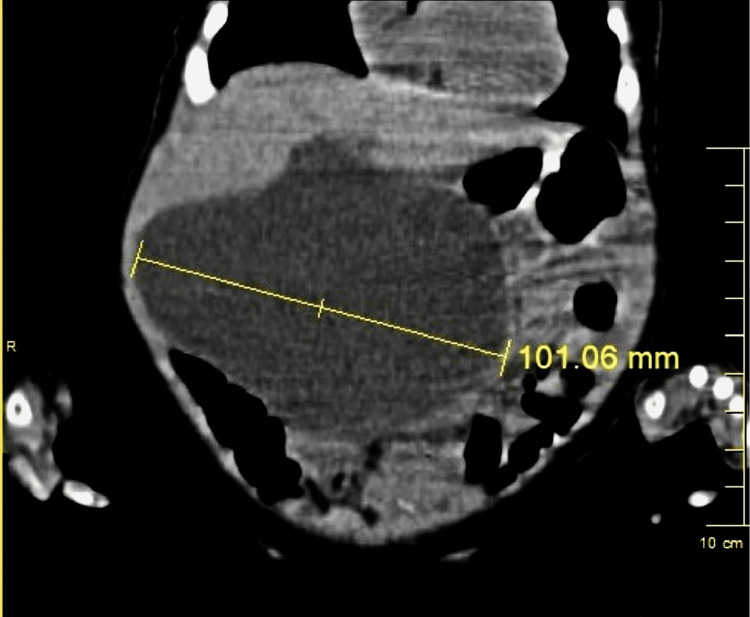
CT images of choledochal cyst measuring more than 10cm at its largest diameter CT: computed tomography

A three-month-old female infant of East Indian descent presented to our emergency department with a three-day history of non-bilious, non-projectile vomiting, and a one-day history of abdominal distention. The mother also described pale, soft stool for three days prior, decreased appetite and increased irritability. The patient did not have any prior history of yellow eye discolouration, dark urine, fever, apparent abdominal pain, or any change in the type of feed being given. At the time, she was both breast- and formula-fed. She was delivered at term via normal spontaneous vaginal delivery with no neonatal intensive care unit (NICU) stay and no history of neonatal jaundice. Family history was negative for congenital hepatobiliary diseases. No abnormalities were observed during her well-baby check-ups. On examination, pulse, blood pressure, oxygen saturation (SPO2) and temperature were all within normal ranges and the patient appeared anicteric. Respiratory and cardiovascular examinations were unremarkable. She however had a large palpable right upper quadrant abdominal mass which was non-tender. Ultrasound showed a 7.2cm right upper quadrant cystic mass extending to the left upper quadrant suggestive of a choledochal cyst. A subsequent CT scan showed an 8.2cm x 10.1cm x 7.3cm choledochal cyst (Figure 1).

Liver function tests (LFTs) were deranged with a direct hyperbilirubinaemia and haemoglobin (Hb) was 9.8g/dl (Table 1). Prothrombin time/ partial thromboplastin time/ international normalized ratio (PT/PTT/INR) were within normal ranges.

**Table 1:** Pre- and postoperative comparison of blood parameters. Hb: haemoglobin; WBC: white blood cells; AST: aspartate transaminase; ALT: alanine transaminase; GGT: gamma-glutamyl transferase; ALP: alkaline phosphatase; g/dL: grams per decilitre; uL: microlitre; mg/dL: milligrams per decilitre; IU/L: international units per litre; U/L: units per litre

Blood investigation	Result (first admission)	Result (Post-op Day 5)	Post-op (3 years)	Reference Ranges
Hb (g/dL)	9.8	11.5	12.3	9.5-13.5
WBC (10^3 uL)	4.74	14.9	10.51	8-20
Platelet (10^3uL)	371	312	275	150-400
Creatinine (mg/dL)	0.2	0.2	0.2	0.5-1.0
AST (IU/L)	6	76.2	37.4	5-40
ALT (IU/L)	65.8	84.6	39.9	5-41
GGT (U/L)	127.7	39	16	5-36
ALP (U/L)	605	204	220	35-104
Direct bilirubin (mg/dL)	3.32	0.12	0.08	0-0.40
Indirect bilirubin (mg/dL)	1.45	0.10	0.10	0-1
Total bilirubin (mg/dL)	1.87	0.22	0.18	0-1.20

**Figure 2:**
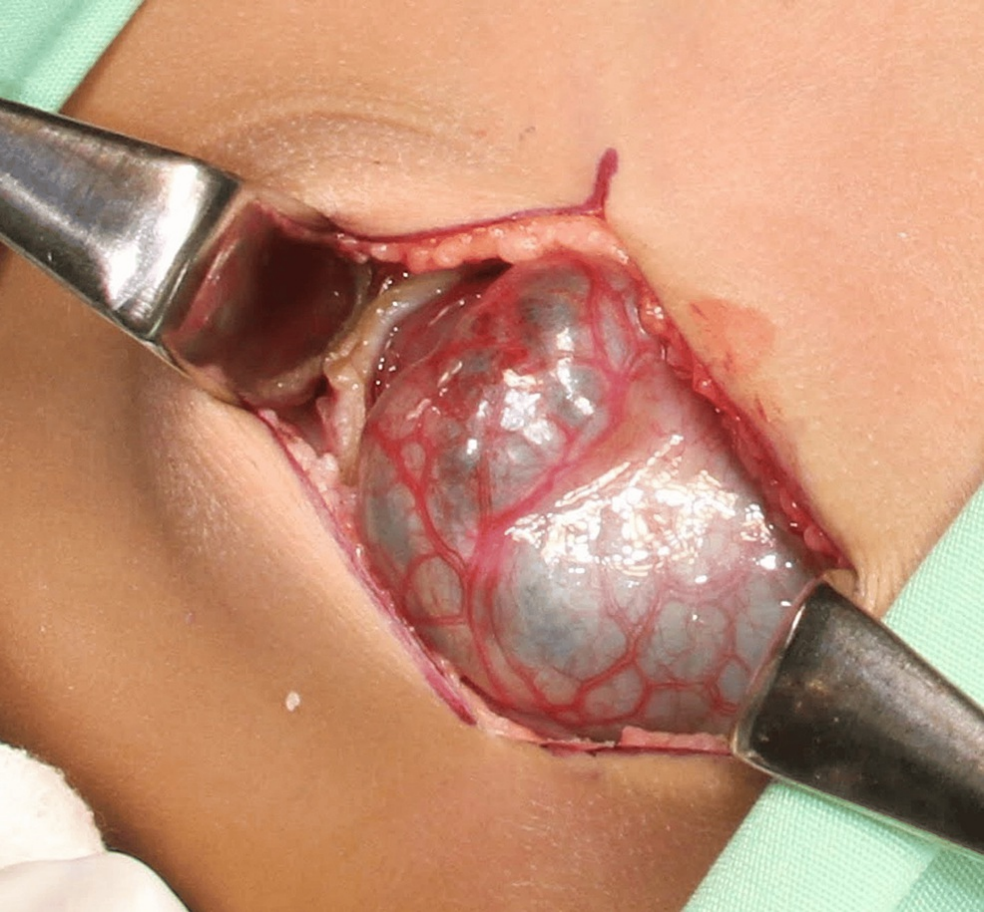
Intra-operative view of choledochal cyst

The patient subsequently underwent open cholecystectomy with excision of the choledochal cyst and hepaticojejunosotomy, using approximately 20cm of bowel to create the Roux-en-Y anastomosis. Intra-operative findings included a giant choledochal cyst (Figure 2) adherent to the postero-inferior aspect of the liver, small bowel, and the head of the pancreas. Intraoperative aspiration of the cyst drained 325ml of bile. The gallbladder was noted to be enlarged and sludge-filled, with a narrow common bile duct (CBD).

The patient remained intubated postoperatively with minimal serous fluid noted from the abdominal drain. Unfortunately, she had a peri-arrest episode on Day 2 postoperatively with subsequent right pneumothorax after resuscitation. Needle decompression was done, and the patient had a return of spontaneous circulation after one round of cardiopulmonary resuscitation and adrenaline. Postoperatively, the bilirubin and liver enzymes began trending down towards normal values (Table 1). The patient was extubated on the third postoperative day, started on oral feeds on day four, and discharged on postoperative Day 12. Histology of the cyst showed thickened and fibrotic walls lined by columnar epithelium. These features were consistent with a choledochal cyst. No evidence of dysplasia or malignancy was detected. This patient is currently being followed up in the paediatric surgery outpatient clinic where her liver enzymes have returned to normal, and she has had nil complications thus far.

## Discussion

Choledochal cysts, first described in 1723, are rare congenital cystic dilatations of the biliary tree [1]. They are commonly found among East Asian children and are more common in female infants [2]. More than 60% present within the first year of life, with an incidence of up to 1:1000 in Asia. In the Western population, however, the incidence is much rarer, at approximately 1:100000 live births [3].

The aetiology remains unclear and is thought to be due to an anomalous communication between the CBD and the pancreatic duct. This causes a reflux of pancreatic secretions into the CBD, resulting in inflammation, epithelialization and weakening of the ductal wall, leading to cyst formation [4]. Another proposed mechanism involves an abnormality of ganglion cells in the slender portion of the CBD. Theories involving abnormal sphincter of Oddi spasm have also been proposed as being related to choledochal cyst formation [4].

Choledochal cysts were classified by Todani into 5 types. The most common type of choledochal cyst is Type 1 (50-80% incidence) [3]. Type 1 choledochal cysts can be further classified into 1A (diffuse cystic dilatation of extrahepatic bile ducts), Type 1B (focal, segmental, cystic extrahepatic duct dilation) and Type 1C (fusiform dilatation, usually from the pancreaticobiliary junction to the intrahepatic duct). Type 2 (2% incidence) involves diverticular dilatation along the extrahepatic duct. Type 3 (1.4-4.5% incidence), or choledochocoele, is defined as intraduodenal cystic dilatation of the distal CBD. Type IV (15-30% incidence) is split into A (multiple dilatations affecting the intra and extrahepatic ducts) and B (multiple dilatations along the extrahepatic duct only). Caroli’s Disease (Type 5 choledochal cyst) has an incidence of approximately 20% and is confined to the intrahepatic duct with multiple dilatations along its course [3]. Giant choledochal cysts are further defined as cysts exceeding 10cm [5].

Infants may present with abdominal pain, jaundice, and a right upper quadrant mass. This triad is seen in approximately 20% of all cases, with 85% of children having at least two features, as compared to only 25% of adults presenting classically [4]. Features of cholangitis, biliary peritonitis or acute pancreatitis may also be observed. In infancy, an obstructive jaundice picture may also be observed, with pale stool, hepatomegaly, and jaundice, making it difficult to differentiate initially from biliary atresia. Prenatally diagnosed choledochal cysts have a worse outcome associated with a higher predisposition for developing liver fibrosis and portal hypertension shortly after birth. Surgical intervention is recommended within the first two weeks of life as biliary sludge displays early formation. The timing of surgery after diagnosis in asymptomatic neonates, however, is not clearly defined [4].

Imaging is multimodal, involving the use of ultrasound, CT, and magnetic resonance cholangiopancreatography (MRCP). Ultrasound is the best initial imaging modality for choledochal cyst identification as well as for evaluation of the entire intra- and extrahepatic biliary system and gallbladder [4]. Ultrasound can also be used in a therapeutic aspect for percutaneous drainage of large cysts in the case of deranged LFTs and coagulopathy before definitive surgery [3]. It is also useful for follow-up and detection of any residual biliary tree dilation postoperatively [3]. A CT scan may also be used to confirm the diagnosis of choledochal cysts. MRCP, however, is the gold standard of imaging for this clinical entity. Prenatal MRI is another imaging modality used as an adjunct to prenatal ultrasound, reserved for defining abnormal abdominal masses detected on antenatal ultrasound [6].

In the case of giant choledochal cysts (≥10cm), or cysts that cause biliary obstruction leading to coagulopathies and increased surgical risk, ultrasound-guided percutaneous cyst drainage has been effective. This procedure, however, must be followed by a definitive operation when the surgical risk has decreased [7]. Our patient did not require a temporizing procedure and an open, on-table cyst aspiration was done for decompression to allow for complete excision. 

Giant choledochal cysts, as previously mentioned, are those measuring more than 10cm at their greatest diameter. They are very rare and pose a host of challenges. They tend to be more symptomatic and surgical excision is also more difficult due to their size. Furthermore, the risk of postoperative complications such as biliary leakage, pancreatic leakage, fistula, and pancreatitis is increased [8].

Surgical intervention is the mainstay of treatment to excise the cyst and re-establish the successful flow of bile into the small bowel, thus preventing infection and malignant development within the abnormal cyst. Treatment for choledochal cysts varies based on type. Type 1 cysts require excision with hepaticoenterostomy, with Roux-en-Y anastomosis being the gold standard. Diverticulectomy is now performed for Type 2 cysts. Endoscopic transduodenal excision and sphincteroplasty are recommended for Type 3 cysts with Type 4 requiring cyst excision and hepaticoenterostomy. Liver transplant and partial resection are the options available for Type 5 disease [9].

Hepaticojejunostomy or hepaticoduodenostomy may be done to re-establish bile flow. The benefits of hepaticojejunostomy include possible lower tension in the Roux limb, therefore decreasing the chance of an anastomotic leak and the diversion of enteric contents away from the biliary tree, hence decreasing the risk of cholangitis development [4]. The risks, however, include prolonged operating time due to the need to fashion the anastomoses and a long defunctioned length of jejunum due to the Roux-en-Y limb. Further early complications include anastomotic leak, fluid collection in the gallbladder fossa and cholangitis. Late complications may include stricture formation, biliary stones and cholangitis [4].

Hepaticoduodenostomy has the benefits of shorter operative time, faster bowel recovery due to less manipulation and shorter hospital stay. There is, however, a higher chance of reflux which may predispose to cholangitis, anastomotic stricture and, rarely, carcinoma. Hepaticoduodenostomy is a more physiological method compared to hepaticojejunostomy and a faster procedure [4].

Prognosis is usually favourable once cyst resection is done early, however, a long-term risk of developing cholangiocarcinoma still exists [9]. Earlier detection and surgical intervention decrease complications such as biliary fibrosis and cirrhosis, therefore surgical intervention should not be delayed [6].

## Conclusions

Giant choledochal cysts are very rare congenital bile duct abnormalities, and none of this size thus far has been recorded in the Caribbean. There is little documentation of choledochal cysts greater than 10cm and these should be considered in the differential diagnosis of a child presenting with an abdominal mass. Early detection and diagnosis are essential to improve recovery and survival rates. Imaging is crucial to the diagnosis of giant choledochal cysts and their differentiation from other causes of abdominal masses. Surgery, based on the Todani type, is the mainstay of treatment, with favourable outcomes post-resection. This case report serves to highlight the occurrence of such a rare congenital abnormality, our management and the subsequent postoperative outcome in a low-resource setting.
